# Effect of intrinsic and extrinsic factors on the pharmacokinetics of TMC278 in antiretroviral-naïve, HIV-1-infected patients in ECHO and THRIVE

**DOI:** 10.1186/1758-2652-13-S4-P186

**Published:** 2010-11-08

**Authors:** HM Crauwels, E van Schaick, RPG van Heeswijk, S Vanveggel, K Boven, P Vis

**Affiliations:** 1 Tibotec BVBA, Beerse, Belgium; 2 Exprimo NV, Mechelen, Belgium; 3 Tibotec Inc, Titusville, NJ, USA

## Purpose of the study

The current analysis examined the pharmacokinetics of the next-generation investigational NNRTI, TMC278, in the pooled double-blind Phase III trials ECHO (NCT00540449) and THRIVE (NCT00543725) in ARV-naïve, HIV-1-infected adults [1] and explored the influence of intrinsic and extrinsic factors on the pharmacokinetic parameters.

## Methods

A total of 1368 patients (24% female) were randomised (1:1) to either TMC278 25 mg q.d. or EFV 600 mg q.d., in combination with TDF/FTC (ECHO) or a choice of either TDF/FTC or AZT/3TC or ABC/3TC (THRIVE). The pharmacokinetics of TMC278 were best described by a two-compartment disposition model in which absorption was characterised by a lag time followed by a sequential zero- and first-order process. Individual values for TMC278 trough plasma concentrations (C_trough_) and area under the plasma concentration-time profile over the dosing interval (AUC_24h_) were estimated from sparse pharmacokinetic sampling in 679 patients in the TMC278 treatment group (8 samples/48 weeks/patient) using the population pharmacokinetic model. In addition, the potential relationship between selected covariates and the TMC278 apparent oral clearance was evaluated.

## Results

There were no differences in the pharmacokinetics of TMC278 between the two trials. The mean (SD) TMC278 C_trough_ and AUC_24h_ for the pooled trials were 80.0 (36.5) ng/mL and 2397 (1032) ng*h/mL, respectively. The apparent oral clearance of TMC278 was estimated to be 11.8 L/h (inter-individual variability 39%) and the apparent volume of the central compartment was estimated to be 152 L (inter-individual variability 117%). The exposure to TMC278 was not influenced by N(t)RTI background medication, age, bodyweight, BMI, estimated glomerular filtration rate and hepatitis B and/or C co-infection status. Gender and race had a statistically significant effect on the TMC278 apparent oral clearance, resulting in a slightly lower apparent oral clearance (and thus higher AUC_24h_) in females (13.6% lower clearance), and in Asian patients (17.2% lower clearance). These small effects had little impact on the overall inter-individual variability in apparent oral clearance and are considered not to be of clinical relevance.

## Conclusions

A population pharmacokinetic model was developed, describing the pharmacokinetics of TMC278 in ARV-naïve, HIV-1-infected adults receiving TMC278 25 mg q.d. No covariates with a clinically relevant effect on exposure to TMC278 were identified.
